# An Overview of Innovative Approaches to Support Timely and Agile Health Communication Research and Practice

**DOI:** 10.3390/ijerph192215073

**Published:** 2022-11-16

**Authors:** Anna Gaysynsky, Kathryn Heley, Wen-Ying Sylvia Chou

**Affiliations:** 1 Health Communication and Informatics Research Branch, Behavioral Research Program, Division of Cancer Control and Population Sciences, National Cancer Institute, Rockville, MD 20850, USA; 2 ICF Next, ICF, Rockville, MD 20850, USA

**Keywords:** health communication, methods, social media, digital health

## Abstract

Innovative approaches are needed to make health communication research and practice more timely, responsive, and effective in a rapidly changing information ecosystem. In this paper we provide an overview of strategies that can enhance the delivery and effectiveness of health communication campaigns and interventions, as well as research approaches that can generate useful data and insights for decisionmakers and campaign designers, thereby reducing the research-to-practice gap. The discussion focuses on the following approaches: digital segmentation and microtargeting, social media influencer campaigns, recommender systems, adaptive interventions, A/B testing, efficient message testing protocols, rapid cycle iterative message testing, megastudies, and agent-based modeling. For each method highlighted, we also outline important practical and ethical considerations for utilizing the approach in the context of health communication research and practice, including issues related to transparency, privacy, equity, and potential for harm.

## 1. Introduction

Much has been written regarding the various communication failures that occurred during the COVID-19 pandemic, including the lack of clear and consistent messaging and the politicization of public health recommendations [1]. However, it is also clear that those in public health and communication were generally unprepared to mount the kind of rapid and agile response needed in a context characterized by the rapid spread of staggering amounts of health information, misinformation, and disinformation facilitated by various digital communication platforms. Standard health communication approaches have proven inadequate for addressing these emerging challenges, and novel, interdisciplinary methods are needed to move the field forward.

Additionally, although the pandemic clearly illustrated the need for new approaches to health crisis communication that are suited to the realities of the evolving information environment, innovations are needed in the field more broadly to ensure that accurate and useful information is effectively delivered to the right person in the right place at the right time. The widespread use of new communication technologies has transformed the way people acquire information and a paradigm shift in health communication is needed to keep pace with these changes. In this article, we discuss several innovative approaches that have the potential to make health communication research and practice more timely, agile, responsive, and precise, thereby increasing the impact of communication efforts in the current information landscape. Even though some of these methods have been used by other fields (e.g., advertising) for many years, they have not yet been widely applied in the context of health communication and are therefore novel for the field—bringing to bear tools from other disciplines such as marketing, computer science, and behavioral economics could help revitalize health communication efforts and open new avenues for advancing health communication research. For each approach highlighted, we outline core components and processes, present specific examples of their application in the health context, and discuss ethical and practical considerations for leveraging these innovative methods for health communication research and practice. Although several of the methods described are most relevant to online health communication efforts and digital health interventions, many of these tools can be used to complement or enhance traditional communication efforts as well, for example by more rapidly identifying effective messages. Our hope is that this overview will serve as a useful reference for health communication researchers and practitioners, and encourage those in the field to integrate a broader range of methods in their work while being mindful of potential pitfalls, such as the possibility that technology-based approaches might exacerbate disparities in certain populations.

## 2. Approaches to Make Health Communication Efforts More Precise, Agile, and Effective

### 2.1. Digital Segmentation and Microtargeting

Market segmentation—or the identification of discrete groups who have distinct needs, characteristics, or behaviors and therefore might require different products, messages, or marketing approaches—has been widely applied in social marketing to achieve various public health and health communication objectives [2]. The proliferation of digital technology over the past two decades has enabled a far more granular approach to segmentation and targeting than was previously possible. Digital platforms, like Facebook and Google, allow audiences to be targeted based on multiple dimensions, including their interests, demographic characteristics, social networks, location, and online behaviors [3,4].

Although the use of digital segmentation and microtargeting for public health is still in its early stages, there is preliminary evidence supporting the effectiveness of these approaches [2]. For example, Morrison et al. developed three short skin cancer prevention videos based on existing literature and focus groups with young women who use tanning beds and placed these videos as advertisements on Facebook for 10 days [5]. The ads targeted women aged 18–34 in six states with high rates of tanning bed use and weak indoor tanning legislation (Alabama, Alaska, Arkansas, Georgia, Kentucky, and Tennessee) [5]. The videos reached 1.25 million individuals and received 1288 comments, 11,415 reactions, and 4201 shares, indicating that social media advertising is a feasible way to reach and engage individuals within a specific demographic and can facilitate more targeted public health campaigns compared to traditional media outlets [5].

An effort that used geotargeting to reach a high-need population offers a different example of how microtargeting through social media can be used for public health communication. In this project, short video messages featuring racially diverse physicians discussing the safety and efficacy of COVID-19 vaccines were placed as advertisements on Facebook and targeted to U.S. zip codes that had (1) the highest COVID-19 death rates, and (2) majority Black or Latinx populations [6]. Like the pilot study by Morrison et al. described above, this campaign, which ran for approximately three weeks, achieved considerable reach and engagement, with the ads reaching nearly 10 million individuals and generating over 10,000 reactions [6].

In addition to location and demographics, online behavior such as internet searches can be used to target messages to a specific audience that is already looking for information related to a particular topic. For example, Serrano et al. used Google AdWords to disseminate skin cancer prevention messages to users who were searching tanning-related key words (e.g., “tanning salon”) [7]. A Truth Initiative campaign offers another example of how digital behavior can be used for segmentation: in this study, researchers divided the audience based on the percentage of an online video they watched in order to determine strategies that would most effectively result in conversion (i.e., follow-through on actions such as visiting the campaign website or posting to social media) [2].

Campaigns can also use a combination of these strategies to reach their desired audience. For example, a gestational weight gain digital campaign conducted in Alberta, Canada used both search engine marketing through Google AdWords and social media advertising through Facebook to reach individuals in Alberta who were either specifically searching for information related to pregnancy weight or who may not have been actively looking for information on the topic but matched the desired demographic and psychographic profile (i.e., female, aged 18–44 years, with an interest in pregnancy) [8]. The complementary strategies both generated a large number of impressions and clicks, and achieved relatively high click-through rates (5.80% for Google Ads and 1.88% for Facebook, which may reflect the fact that the Google Ads were shown to users who were already interested in the topic) [8].

As public health efforts increasingly rely on digital channels to reach audiences, additional research could help optimize targeting in the service of health promotion. A large set of predictive factors, including more traditional segmentation characteristics such as demographics as well as digital behaviors, could be explored in order to evaluate their utility in enhancing campaign engagement among target populations. Additionally, further research is needed to assess the impact of efforts that leverage digital segmentation and microtargeting on real-world outcomes, such as behavior change. To date, most work on this topic has been limited to using measures of online engagement to evaluate impact; research generating evidence of effectiveness for additional outcomes, such as cognitions, attitudes, decisions, and behaviors, could help make the case for more widespread use of these approaches in practice.

#### Ethical and Practical Considerations

Although the examples highlighted above suggest that digital segmentation and microtargeting hold promise, practitioners seeking to leverage these strategies for health communication efforts should be aware of several important caveats. First, strategies that rely on targeted advertising tools offered by commercial platforms will need to contend with the limitations of these tools. For example, these tools can only reach the userbase of a given platform—which may be large, but is not necessarily representative [4]. Social media platforms are a good channel for engaging with some harder-to-reach groups (e.g., young people from certain racial/ethnic minority populations who use social media at higher rates), but it is important to remember that there may be groups who are not online or who do not use the specific platform being utilized, and therefore may be missed by campaigns that rely on social media. Furthermore, because microtargeting only reaches specific segments of a given platform’s users, this practice has the potential to undermine equal access to information and interventions if not implemented carefully [9].

Campaigns relying on ad targeting tools must also comply with a given platform’s policies, which can limit the types of campaigns that can be conducted and may be subject to change with little notice. For example, in 2016, Reiter et al. used Facebook ads to recruit young gay and bisexual men into a human papillomavirus (HPV) vaccination intervention using LGBT-related key terms generated from users’ timelines and “liked” pages to identify members of the target population [10]. Based on the results of their study, the authors concluded that Facebook ads could be a cost-efficient strategy for reaching young gay and bisexual men [10]. However, in 2021, Facebook announced that it would be removing specific targeting options that relate to interest in “sensitive topics”, including sexual orientation [11], meaning a similar outreach effort to young gay and bisexual men would not be possible today. Even though these policy changes could make it more difficult for public health practitioners to target certain priority populations for health promotion, they were enacted with the aim of protecting potentially vulnerable populations from harm, which is important as these powerful microtargeting capabilities can also be abused (e.g., allowing advertisers to prevent members of certain groups from seeing ads for housing or employment [12]).

Another issue is that there is a lack of control both on the part of campaign managers and users when social media platforms are used for communication. For example, campaigns are not able to assess distress in response to their ads and individuals may lack the ability or the knowledge needed to control the ads they see, which could lead to unintended consequences. For example, being exposed to messages providing information about having a healthy pregnancy could be distressing to an individual who recently experienced a miscarriage, but there may not be proper safeguards in place on social media platforms to protect that individual from being exposed to this content (at least by default) or to address their distress once they are exposed. Furthermore, users may not always engage with social media messages in a way that is consistent with campaign goals. For example, they may disagree with the message being disseminated, and this counter-messaging could then be visible to other users as well. Concern regarding the potential for negative comments targeting participating healthcare providers and the inadvertent spread of misinformation led de Vere Hunt et al. to disable comments on the COVID-19 vaccine-promotion video ads used in their campaign [6]. Practitioners will need to take such issues into account to ensure that their campaigns do not inadvertently cause harm.

Finally, it is important to consider that many people may not be aware that social media platforms collect information on their characteristics and use such information to target ads—and they may not find this process acceptable once they become aware. In 2019, a Pew Research Survey asked a representative sample of Facebook users about the information that Facebook collects in order to target ads to them, and found that a majority of users (~74%) were not aware that Facebook collected this information until they were directed to check their “ad preferences” page as part of the study [13], and about half (51%) of the respondents said they were not comfortable with this practice [13]. The overall lack of transparency and opaqueness of the process that enables microtargeting largely leaves users without any indication that they have been targeted for a specific ad, or why [9]. The public’s lack of awareness and concern about these targeting practices raises questions about whether it is appropriate for health communication practitioners to take advantage of these tools, suggesting a need to explore whether individuals find the use of these tools to target health promotion messages, rather than ads for commercial products, more acceptable. Targeting individuals with relevant, timely public health messages requires collecting information about them; however, some ethicists and privacy scholars argue that just because these data exist and are available, does not necessarily mean that they can be used by researchers or practitioners: individuals who share information on social media platforms may have certain expectations about how their data will be used, and the use of these data for message and ad targeting might violate those expectations, even if the use is consistent with applicable rules and terms of service [14].

### 2.2. Social Media Influencer Campaigns

Working with influencers (i.e., individuals who have a high number of followers on social media, either in general or among a specific subpopulation [15]) to disseminate health information is another novel communication strategy that leverages some of the unique features of social media platforms to reach target audiences with effective messaging. Collaborating with influencers who already have an established audience in the target community can enable health messaging to be tailored and disseminated in a way that feels more personally relevant to members of those communities, and takes advantage of the conversational dynamics and social influence embedded in online networks [16]. Although influencers rarely have health-related expertise, some research suggests that they may still be perceived as trusted sources of health information among their followers [17]. Working with influencers could also present health practitioners with an opportunity to bypass filter bubbles and self-curation in order to reach populations that may not have an active interest in health information [16]. Micro-influencers (influential accounts with a relatively small but still significant following on a given platform), could be especially effective messengers as they may be perceived as trusted friends or aspirational peers rather than celebrities [18], and while celebrity influencers may have greater reach overall, micro-influencers tend to garner greater engagement rates on their posts [15].

The use of influencers is still fairly limited in public health practice, but research is beginning to show their potential in the context of health communication [18]. Work conducted by Bonnevie et al. [18] offers a recent example illustrating the successful utilization of influencers for health promotion. In this study, the research team assessed the feasibility of promoting flu vaccination to African American and Hispanic populations through social media influencers, positing that influencers could be more successful at communicating health information to these groups, as they already use the language and style of speech of the target audience [18]. As part of the campaign, influencers were asked to choose from a set of vetted messages about flu vaccination and create their own original, user-generated content in either English or Spanish [18]. The campaign garnered high levels of engagement and demonstrated that campaigns relying on influencers can reach large numbers of people in a relatively inexpensive way [18]. Notably, Spanish-language posts were found to have especially high engagement rates, potentially because Spanish-speaking individuals are less accustomed to seeing health information presented in their native language and in a style that resonates with them, and were therefore more motivated to engage with the content [18]. Post-campaign survey data suggests that the campaign was successful, as respondents in the campaign region who reported having seen a message promoting the flu vaccine on social media were more likely to have received a flu vaccine and to hold positive perceptions of the vaccine compared to those who did not report being exposed to social media messages promoting vaccination [18].

An effort initiated in Finland during the COVID-19 pandemic offers another illustrative example of how influencers can be leveraged to communicate health-related information to the public. The Prime Minister’s Office led a campaign to encourage and support social media influencers in communicating timely information about the coronavirus to their followers by creating an information center for them, sending newsletters, and holding informational webinars [19]. As part of the “Corona Facts” campaign, influencers were asked to post about fact-checked content and use the hashtag #coronafacts [19]. A qualitative content analysis showed that influencers participating in the campaign tended to adapt the messages to their own style, and that their posts demonstrated specific consideration of the particular needs of their followers (for example, family influencers posted about things to do with kids while staying at home, while travel influencers discussed how to find adventure in nearby locations) [19]. The authors point out that posts reflecting the influencer’s own interpretation of the campaign (using their own photos and words) more clearly leverage the factors that make influencers effective, and were likely better able to connect with the intended audience [19].

Influencers were also used in a campaign launched by NYC Health + Hospitals in 2020 to encourage New York City residents to get tested for COVID-19 [20]. Because one of the main goals of the campaign was to reach women, the LGBTQ+ community, racial/ethnic minority populations, and other historically disadvantaged groups, NYC Health + Hospitals and its partner marketing agencies recruited both celebrities (e.g., actor John Leguizamo, ballerina Misty Copeland) and NYC-based micro influencers (e.g., Gerry Isabelle—a Dominican-American travel and lifestyle digital creator) who could effectively engage these target communities [20]. During the five-month campaign, the paid influencers created original content based on guidelines and assets provided by NYC Health + Hospitals to urge New Yorkers to get tested. Examples of efforts focused on special populations included an IGTV (Instagram TV) conversation between DJ and new mom Vashtie Kola and an NYC Health + Hospitals obstetrician-gynecologist regarding COVID-19 prevention and care during pregnancy and post-partum, as well as Facebook Live town halls hosted by NYC-based drag queens targeted to the city’s LGBTQ+ community [20]. Although the campaign faced challenges such as increased media costs due to the 2020 presidential election and changes in social media platforms’ algorithms for sponsored posts, overall the campaign was deemed a success, achieving both wide reach (influencer messages on COVID-19 testing yielded more than 2.4 million impressions) and high engagement rates [20].

Influencer-based campaigns can also complement other communication strategies, such as targeted digital ads. For example, the Food and Drug Administration’s “Fresh Empire” tobacco education campaign used both influencers and paid digital/social media advertisements as part of a multichannel, multilevel communication strategy to reach African American, Hispanic, and Asian American/Pacific Islander youth who identify with hip-hop culture [21]. The objective of the digital ads was to maximize reach and frequency of campaign message exposure on an individual level in order to change tobacco-related knowledge, attitudes, and beliefs, while the goal of the influencer component was to expand campaign reach, improve engagement, and increase message credibility, as well as to spark positive conversations around tobacco prevention in the social environment of the target population [21]. Utilizing influencers to disseminate campaign messages leverages the perceived personal connection followers have with influencers and the trust influencers have cultivated with their audience to increase the perceived authenticity and credibility of a campaign [21]. The influencer strategy was deemed successful based on measures of impressions, reach, and engagement: posts from the six micro-influencers participating in the campaign generated more than 3 million impressions, and influencer content achieved higher engagement rates compared to the campaign’s non-paid content [21].

#### Ethical and Practical Considerations

Relying on individuals who often do not have any background or training in health or medicine to deliver public health messages carries some risks [21]. For example, an influencer could hold views or promote content that conflicts with the goals and values of a campaign [21]. Therefore, influencers need to be carefully vetted before being recruited for participation in a public health campaign and then monitored on an ongoing basis to ensure they are not engaging in behavior that might be detrimental to the campaign. In their study, Bonnevie et al. conducted a 3-month retrospective review of each influencer’s public social media posts to ensure they did not promote harmful products such as alcohol, tobacco, or firearms, or post any inflammatory or offensive content [18]. 

Briefing influencers on the science of a given health topic and providing guidelines for developing content can help to ensure that the scientific accuracy of messages is preserved, even as influencers try to convey messages in their own “voice” [21]. Reviewing campaign-related content before an influencer posts it could also allow public health practitioners to ensure messages are appropriate and aligned with campaign objectives, but this is not always possible (e.g., during a livestream), and it should be recognized that working with influencers can necessitate relinquishing control to a certain degree. It should also be noted that there are examples of successful social media influencers who have backgrounds in health or medicine [22], and there could be opportunities to work more closely with these individuals on health promotion efforts. These influencers could be particularly appealing as messengers since their audience already looks to them and trusts them to provide accurate health information, and their knowledgeability would reduce the risk of messages getting distorted. However, there are currently relatively few health professionals who have achieved “influencer” status, and these accounts may not reach those who are not actively interested in health, so there may be limitations to efforts that only recruit social media influencers with health, medical, or science training. Nevertheless, encouraging more health professionals and scientists to be active on social media and to increase their influence on these channels could be a worthwhile endeavor, with the caveat that effective health communication is a skill that varies widely among individuals and best practices for training health professionals to engage effectively on social media platforms would likely need to be developed. 

Another potential concern is the possibility that participating in a campaign may entail risk for the influencers themselves, particularly when a health topic might be seen as controversial. For example, taking part in a vaccine-promotion campaign could leave influencers vulnerable to harassment from anti-vaccine groups [23]. These consequences should be considered carefully, and training or resources should be offered to influencers that are participating in campaigns that address sensitive subjects. Furthermore, the fit between an influencer’s audience and the health message needs to be carefully considered. It is possible that a particular influencer’s followers would not be receptive to certain health messages (e.g., vaccine promotion, alcohol use reduction), which could not only render the campaign ineffective, but negatively affect the influencer’s relationship with their audience. Of course, campaigns may seek to reach individuals who do not already endorse a health message or engage in a certain health behavior, and working with social media influencers could be an opportunity to achieve that goal, but careful consideration should be given in the case of health messages that might be negatively received by the intended audience, as this could both be ineffective, and have negative consequences for the participating influencer. Influencers should be regarded and treated as partners, and the potential of negative impact on their status or livelihood should be given serious consideration.

Lastly, extra care should be taken when using influencers to reach potentially vulnerable populations. For example, leveraging influencers to reach adolescents may be effective, but if adolescents do not have the adequate health or media literacy skills to critically engage with the content and simply accept what their favorite influencer tells them, the campaign may end up being manipulative rather than informative or persuasive as its designers intended. Businesses exploit the parasocial relationships, admiration, and trust adolescents have toward influencers in order to promote unhealthy products such as vapes, junk food, and plastic surgery procedures—even though using these mechanisms to promote healthy choices is undoubtedly a more noble goal and can generate benefit for the target population, whether the ends justify the means should be carefully considered to ensure that the approach used is not unduly limiting autonomy or circumventing deliberative decision-making processes. Similarly, the use of influencers to reach individuals experiencing specific health conditions also needs to be done thoughtfully. Providing general information about the illness or tips for self-management may be welcome, but promotion of specific treatments, for example, could be more problematic if it affects patient preferences or interferes with the patient’s therapeutic relationship with their provider.

### 2.3. Recommendation Algorithms

In addition to helping practitioners more effectively reach their target audience with a given health message, digital technologies offer innovative ways to identify and select messages or other communication content that would be most effective for a given individual or group. For example, in recent years there has been an increased interest in the possibility of applying recommender systems to health communication. Companies like Amazon and Netflix use recommender systems to make personalized suggestions regarding relevant products and content to consumers [24]. The goal of these algorithms is to predict a target individual’s reaction to items that they have not yet seen based on the new item’s similarity to other items the user has interacted with, the preferences of other similar users, or a combination of these [24]. Although recommender systems have been successfully used in commercial applications, little research to date has explored whether they can be used to effectively recommend health messages to individuals.

Kim et al. attempted to address this question by testing the effectiveness of an algorithm that selects smoking-related public service announcements (PSAs) for smokers in predicting a target smoker’s evaluations of the PSA and encouraging smoking cessation [24]. The algorithm, which incorporated smokers’ PSA rating history, individual differences, content features of the PSAs, and other smokers’ PSA ratings, was tested in a longitudinal online experiment in comparison to two other selection methods (“best in show”, where PSAs most preferred by participants overall were selected, and “off the shelf”, where messages were randomly selected from a pool of eligible PSAs) [24]. Results indicated that the recommender algorithm was able to more accurately predict smokers’ evaluations of perceived message effectiveness than the simple-average method used for the “best in show” approach, and that smokers who viewed PSAs suggested by the recommender algorithm were more likely to find the messages persuasive and report changing their smoking behavior than those who received PSAs chosen at random [24]. The authors suggest that the algorithmic approach to message recommendation is worth additional consideration, as it could offer a more efficient alternative to message tailoring, which is limited by the need to collect substantial amounts of personal information from individuals and the costly and complex process of altering different message features for each individual [24].

Writing in the *Journal of Medical Internet Research*, Sadasivam and colleagues suggest that recommender systems could enhance computer-tailored health communication interventions, and argue that combining theory-based computer tailoring with empirically based recommender systems could be especially effective [25]. Recommender systems can enable tailoring based on a larger number of variables, are not limited to theoretical constructs or researchers’ knowledge (which can be limited), and have the ability to adapt in real time [25]. A combined approach could see users initially segmented according to a pre-determined variable based on theory or existing literature (such as stage-of-change), with the recommender system then further tailoring within each segment based on implicit or explicit feedback collected from users [25]. A study conducted by Sadasivam et al. comparing a standard rules-based system that tailored messages on smoker’s readiness to quit to a novel recommender system that selected messages based on user ratings, showed that the recommender system outperformed the standard tailoring system in the number of days that participants agreed that the selected message influenced them to quit, and yielded higher (but not statistically significant) rates of smoking cessation among participants who completed follow-up assessments [26]. Furthermore, a follow-up study testing the recommender system among African American smokers indicated that this approach could be particularly effective in this population, as their ratings of message influence were higher, they had higher engagement with the intervention, and they were significantly more likely to report cessation behavior compared to white smokers who had participated in the original study [27].

#### Ethical and Practical Considerations

Despite some initial evidence that recommender systems could be a promising strategy for selecting personalized messages, there are some notable challenges to deploying recommender systems in health communication efforts. First, most interventions will likely lack a significant amount of user behavior or feedback data at the outset upon which to build these algorithms, which may necessitate a pre-intervention stage to collect the needed data [25]. Second, due to resource constraints, unlike large commercial companies health interventions usually rely on small samples and work over short time frames, which can limit the amount of data collected during the intervention and threaten the accuracy of the algorithm [25]. Third, unlike assessing the effectiveness of recommendations for products, which can be measured by metrics like purchase decisions, assessing the effectiveness of health messages may be more complex (e.g., a high message rating may not necessarily translate to behavior change) [25]. In fact, it is possible that individuals might not find effective messages to be appealing; for example, fear-based messages may be effective in motivating behavior change under certain circumstances, but people may also find them distressing [28].

The literature on recommender systems also points to several ethical issues for practitioners and researchers to consider, including opacity, bias, and tradeoffs between privacy and recommendation quality [29,30]. The idea that the reasoning behind an algorithmic process should be explained to a user alongside the output (i.e., “explainability”) is often discussed in the context of recommender systems. Explanations that provide transparency into how and why a recommendation was made can increase users’ trust in the system and mitigate the risk of encroaching on their autonomy [30]. However, sometimes algorithms are so complex it is difficult to ascertain or explain how or why they produce a given output, and even when the mechanisms underlying a recommendation are known, how to explain recommendations that might be sensitive (e.g., mental health content) must be handled with care [29]. Furthermore, the fact that explanations may reveal connections between items could pose a privacy risk, as these connections could allow users to be identified [30]. Finally, a more practical concern with explanations is that they may influence user perceptions of the messages or items recommended (e.g., knowing that a message received high ratings from others could shift a user’s attitude toward the message) [31].

Greater transparency into the workings of algorithms could also help uncover potential biases in these systems. Research has highlighted several potential sources of bias in recommender systems. For example, observation bias can result from feedback loops generated by the system’s recommendations [31]. Bias can also result from imbalanced data: the data available to the system may reflect existing social patterns that are discriminatory toward certain groups, leading the system to replicate these social patterns [31].

Another important issue to consider is that in order to achieve greater accuracy, recommender systems require more information about users. In the context of health, the data needed to provide relevant recommendations may be sensitive, and the tradeoff between privacy and personalization may therefore have a greater risk associated with it [29]. Personal data collected to inform the recommender system should be treated as sensitive and stored and protected appropriately [29]. There is also potential for harm when recommender systems lack certain information about the user or their context and then provide recommendations that may be problematic. For example, a recommendation to increase socialization with friends as a way to cope with depression may be detrimental to someone whose friends enable their drug or alcohol use [29]. Therefore, when a system does not have sufficient data to ensure a recommendation will not be harmful, it may be safer to withhold the recommendation. Users should also be warned that recommendations may not always be correct [29].

Lastly, there is concern that a recommender system primarily based on user feedback may replicate already existing counterproductive beliefs or biases in the target population. For example, research has demonstrated lower rates of nicotine replacement therapy (NRT) use among African American smokers due to negative beliefs regarding the safety and addictive potential of NRT, among other factors [27]. A smoking cessation recommender system may therefore not suggest messages promoting NRT to an African American individual (which could be helpful to their cessation attempts) if other users in this population have provided low ratings for these messages or avoided interacting with them [27].

### 2.4. Adaptive Interventions

Digital technologies, such as mobile phones and wearable devices, can enable timely and precise health communication interventions by facilitating the collection of behavioral and contextual data needed for the delivery of just-in-time adaptive interventions (JITAIs), which adjust the timing and content of intervention components to better meet an individual’s needs and circumstances [32]. These technologies can collect self-reported data (e.g., current mood) through brief assessments as well as objective behavioral data such as level of physical activity from built-in accelerometers, while geolocation data from these devices can enable inferences to be made about a user’s context (e.g., local weather, proximity to tobacco retailers) that can further inform intervention strategies [32]. Additionally, because many mobile devices (such as smartphones and wearables) are almost constantly with the user, JITAIs delivered through these technologies can reach people when they are most receptive to, or most in need of, the intervention [33]. For example, Dorsch et al. developed a contextual just-in-time adaptive mobile intervention to reduce dietary sodium intake among patients with hypertension, which uses geofencing to detect when a participant is entering a restaurant or a grocery store and, based on this information, provides (1) a just-in-time push notification message to promote behavior change, tailored on the location (grocery store or restaurant), the participants’ top high sodium foods, and their confidence in following a low-sodium diet, and (2) the ability to easily identify lower-sodium options available at the given location [34,35]. Results of a pilot randomized control trial showed that the intervention led to a greater reduction in dietary sodium intake in adults with hypertension compared to a control group over an 8-week period, as measured by 24-hour urinary sodium excretion [35].

The foundational features of JITAIs are the decision rules that define when, where, and how an intervention component should be delivered (e.g., a prompt to take a walk around the neighborhood should be delivered only when a person is (a) located inside their home, and (b) the weather is suitable for walking outside) [33,36]. These decision rules can be tested and optimized through micro-randomized trials, where the delivery of a specific intervention component is randomized at certain decision points on the basis of specific tailoring variables. An outcome is then measured after each decision point, both when the intervention component is delivered and when it is not, to assess the impact of delivering the intervention component at specific decision points and to test if intervention components work only in certain situations (e.g., only when an individual is at home on a weekend day) [33].

More recently, there has been a growing interest in continuous-tuning interventions, which may make JITAIs more precise by adjusting the intervention through real-time optimization algorithms based on a specific user’s own data, rather than predetermined decision rules based on the results of previous trials [33]. N-of-1 methods can be used to systematically account for individual differences (e.g., different variables may be more or less predictive of a specific behavior for different individuals), thereby improving intervention effectiveness for each participant [33]. For example, in a proof-of-concept study, Conroy et al. used a control systems engineering approach to develop personalized models of behavioral responses to an intensive text message-based intervention [37]. In this study, 10 adults who did not meet federally recommended levels of physical activity received five text messages daily at random times for 16 weeks and had their activity levels assessed using activPAL3 activity monitors. Messages were randomly selected from three different categories: “move more”, “sit less”, and “general facts/trivia”. A dynamical systems model was estimated for each participant to examine the magnitude and timing of response to each type of text message. The results revealed heterogeneous responses to different message types that varied between people and between weekdays and weekends, indicating potential value in applying personalized message selection algorithms [37]. To illustrate, the authors compared the cumulative response curves of two different participants: for one participant, weekend stepping time increased following “move more” messages, but not in response to “sit less” messages or general facts/trivia, whereas for the second participant, weekend movement time increased following “sit less” messages but not in response to “move more” messages or general facts/trivia, suggesting different messaging strategies would be needed for these participants. Additionally, patterns that emerge across the individual models can inform understanding of behavioral responses to the intervention more generally (e.g., in this study, half of the sample had a greater behavioral response to text messages on weekends, while the other half had similar behavioral responses to text messages on weekends and weekdays) [37].

Although the model used in this study was relatively simplistic (e.g., it did not account for many relevant contextual factors such as the weather or the individual’s location), and the message features tested were relatively rudimentary (i.e., whether they encouraged more physical activity or less sedentary activity), additional work testing different communication strategies in different contexts (e.g., whether individual responses to gain vs. loss-framed messages when feeling angry vs. fearful differs between individuals), as well as different modes and formats (e.g., text vs. video), could help advance the field and enhance the effectiveness of health communication interventions. Furthermore, because these types of approaches are still very new, especially in the context of health communication interventions, their feasibility needs to be evaluated with larger and more diverse samples and tested against other approaches (e.g., message selection based on user-reported preferences) to assess whether they outperform less complex approaches to customizing content [37].

It should also be noted that N-of-1 designs are not always suitable approaches for a given topic or intervention. For example, because they require variability in the predictors and outcomes of interest, they tend to be a better fit for complex behaviors and health issues that are frequent, dynamic, and idiosyncratic—such as physical activity, alcohol intake, and smoking [33,38]. They are also better suited for time-specific intervention techniques (e.g., cues/prompts, daily goal setting), and less appropriate for approaches that may have a significant carry over effect, such as education or skill building [38,39].

#### Ethical and Practical Considerations

As of 2021, approximately 15% of Americans do not own a smartphone [40], and this would likely affect their ability to benefit from adaptive interventions, which tend to rely on smartphones or other mobile devices for delivery [32]. Since underserved and vulnerable groups, such as older individuals and those with lower income and educational attainment, also have lower rates of smartphone ownership [40], issues of equity need to be considered when deploying smartphone-based interventions over more accessible interventions, such as those that can be delivered over standard cellphones.

Additionally, many adaptive interventions collect large amounts of passive data from participants’ devices, making it important to ensure that individuals fully understand what data are being collected and how they are used [33]. Ensuring informed consent is especially important when working with populations who may have lower levels of digital literacy (e.g., older individuals) [33]. Allowing users to have more control over their personal data and allowing them to decide what types of data are collected could also increase the perceived trustworthiness of the intervention [29], though any trade-offs in functionality or accuracy as a result of less data being collected would need to be clearly explained. Furthermore, while some have argued for a user-centered approach that provides individuals with tools to enable them to explicitly control the way their data are collected and used in order to increase transparency and autonomy, it should be noted that a drawback to such an approach is that it places the burden on the user [31]. This could be problematic as it means the level of data protection received would vary based on each individual user’s own awareness and level of technological understanding [31]—and may or may not reflect their actual preferences. Given the large volume of potentially sensitive personal data that is being collected, public health practitioners and researchers also need to ensure that they have appropriate policies and structures in place regarding data transmission and storage to adequately protect these data [33].

Lastly, safety must also be considered when designing adaptive health communication interventions. It may not be appropriate to intervene in certain situations (e.g., delivering a push notification when an individual is driving), or it may be necessary to modify the intervention delivered based on the user’s circumstances (e.g., in a mental health intervention, indications of suicidal thinking would need to trigger a call from a counselor rather than an automated message) [41].

## 3. Approaches to Make Health Communication Research More Timely and Useful

### 3.1. A/B Testing

Digital platforms offer researchers a rapid, low-cost way to test messages in order to better inform health communication efforts [42]. Specifically, the appeal of different message features for a target audience can be assessed by conducting A/B testing (also known as split-testing). A/B tests are controlled experiments, usually conducted online, where two versions of a given variable are compared to identify the most effective outcome [43].

A/B testing can be conducted using the same targeted advertising tools offered by social media companies for digital segmentation and microtargeting. An A/B testing feature is available on several platforms including Facebook and TikTok; however, it is still possible to conduct A/B testing on platforms, like Twitter, that do not offer the feature by creating two different sets of messages for the same audience [4]. In A/B testing, the audience is divided into random, non-overlapping groups and the performance (or cost) of each ad is tracked [44]. Differences in performance metrics (e.g., click-through-rates, likes, shares) can then be compared between different versions of the campaign being tested to see which one is preferred by the target audience; it is also possible to compare the performance of different versions of the campaign in different target audience segments to see which version is more suitable for a specific population [4]. For example, researchers in South Carolina used split-testing to assess the performance of identical HPV vaccine messages paired with either a photograph or an abstract graphic, finding that photographs outperformed graphics for all messages [44]. Meanwhile, a campaign promoting safe fish consumption among women used A/B testing to assess different message features and to see whether message preferences differed between pregnant and non-pregnant women—two distinct audience segments within the target population [42]. The results suggested that, overall, women preferred a question format (vs. statement format) for the message, marketing (vs. patient education) copy, and uncertain text that used words like “can” and “may” (vs. certain text), but that pregnant women were more likely to prefer a message invoking experts while nonpregnant women preferred a message invoking physicians [42]. Results from these types of tests can be used to guide decisions prior to launching a larger-scale campaign.

Aside from the capabilities offered by social media ad tools, A/B testing can also be performed using other platforms, such as crowdsourcing sites, which can allow for greater flexibility (e.g., in the type of materials or outcomes assessed). For example, Fan et al. used Amazon Mechanical Turk (MTurk) to identify the best length and content for postoperative instructions after autologous breast reconstruction through staggered A/B testing that compared different versions of patient education materials [43]. First, participant quiz scores were compared after being exposed to materials written at a sixth-grade vs. thirteenth-grade level; in the next round, materials of varying lengths (written at the grade level that yielded the highest quiz scores in the previous experiment) were compared (400 vs. 800 vs. 2000 words), and finally, quiz scores after participants were provided the best performing text with or without pictures were assessed [43]. The experiments indicated that average quiz score was significantly higher for the materials written at the sixth-grade level, and for materials limited to either 400 or 800 words [43]. Scores were not significantly different between the group that saw materials with pictures and the group that saw materials without pictures [43]. Findings from these types of experiments can guide the development of more effective patient education materials for real world uses.

In addition to online environments, A/B testing could also be used to improve communication tools in the clinical setting. For example, a research team at NYU Langone Health used A/B testing as a strategy to improve clinical decision support features in their medical center’s electronic health record system [45]. The team tested three different versions of a tobacco cessation alert that varied in the framing of the message: financial (emphasizing the additional revenue that a physician could generate by performing tobacco cessation counseling) vs. evidence-based (highlighting that tobacco cessation was part of providing high-quality care) vs. regulatory (stressing that tobacco cessation counseling was integral to the institution’s expectations and policies), and in an additional round of testing, assessed the impact of adding images to reinforce the message framing [45]. The experiments indicated that neither the framing nor the addition of images resulted in significant differences in acceptance rates for the tobacco alert, possibly because changes to alert presentation might not be sufficient to modify clinical practice behaviors in a significant way [45]. Nonetheless, A/B testing could enable efficient, rapid, and rigorous evaluation of clinic-based communication tools, thereby helping to improve their efficacy and/or preventing clinics from wasting resources on ineffective initiatives [45,46].

#### Ethical and Practical Considerations

When social media platforms are used to conduct A/B testing, users are not asked to provide consent (and are rarely even made aware of the experiment), and no protections exist for the “human subjects” that are taking part in this type of testing [47]. The risk posed by A/B testing is usually minimal, since it is generally used to compare different types of messages or message format features; however, researchers should take the lack of participant consent into account, especially for sensitive topics or if they are planning more advanced forms of behavioral experimentation or to test strategies that might be considered deceptive or manipulative. In those cases, especially, it may be preferable to use platforms that the researcher has more control over (e.g., a website designed for the study), so that either informed consent can be obtained, or, when advanced disclosure might confound an experiment, debriefing can take place at the end [48].

More generally, some work suggests that people may be uncomfortable with the idea of A/B testing even in less controversial situations. Meyer et al. write about the “A/B illusion”, or the observation that individuals appear to object to randomized experiments comparing two unobjectionable policies or treatments (neither of which are necessarily known to be superior), but do not seem to take issue with the idea of either one of the conditions being universally implemented, even when in both cases (the experiment or the universal application), informed consent would not be obtained and therefore there would be no difference with respect to autonomy [49]. In a series of studies, they show that people frequently rate experiments designed to establish comparative effectiveness of two conditions as inappropriate, even when the individual treatments being compared are widely seen as appropriate (e.g., providing a checklist on a doctor’s badge vs. on a poster in the operating room) [49]. The authors note that although it is reasonable to want the opportunity to consent, an A/B test should not be seen as more ethically problematic than a decision to universally implement either of the untested options when neither of the two treatments is objectionable or known to be more effective [49]. The public’s negative perception of randomized experiments may prevent decision makers from running large-scale field experiments out of concern that they will face backlash (which may lead them to select one of the options to implement based on assumptions rather than data), and may also prevent individuals from participating in these types of studies [49]. Negative attitudes toward randomized experiments may reflect a lack of understanding as well as a heuristic perception of experiments as being inherently risky [49]. In order to implement A/B testing in support of health communication on a wider scale, it may first be necessary to better educate the public about these types of experiments and to err on the side of greater transparency and consent, even when experiments entail limited risk.

### 3.2. Efficient Message Testing Protocols

In addition to A/B testing, other methods have also been proposed to allow candidate messages to be evaluated more efficiently. For example, Kim & Capella propose a rapid message testing protocol that yields valid and reliable message evaluation results, thereby allowing campaign developers to assess messages quickly and efficiently and reducing the time and resources required for pre-testing [50]. The authors point out that efficient procedures focused on likely persuasiveness of a large set of messages are needed to complement “gold standard” message evaluation methods that focus on long-term behavioral outcomes from a small set of messages tested in control and comparison groups [50]. Campaigns need information regarding the effectiveness of messages before deploying a campaign and testing actual message effectiveness in the field, and the protocol developed by Kim & Cappella utilizes measures of perceived argument strength (PAS) and perceived message effectiveness (PME) to assess a large number of candidate messages quickly and produce a rank order of their potential to create acceptance [50].

Following this protocol, a sample of individuals from the population being targeted by the campaign is recruited and assigned to evaluate a random set of messages from a pool of candidate messages (presented in random order), rating them on PAS and/or PME using simple, validated measures [50]. Having each evaluator rate multiple messages improves efficiency by increasing the number of evaluations per message to ensure stability of estimates, without increasing the sample size [50]. Additionally, the double randomization (random selection and random ordering) used in the protocol design helps improve inference and prevent bias when message scores are aggregated [50]. Kim & Cappella also note that it is important to include multiple messages that represent an underlying construct in the message pool in order to avoid the “case-category confound”; testing several messages that represent the same construct but vary in other ways increases the likelihood that the observed effect is due to the construct being tested and not being driven by an irrelevant factor [50]. Using a large pool of messages that display variability in message features also allows clearer distinctions to be made between stronger and weaker versions of a message feature [51].

Another approach to message testing, based on ecological momentary assessment (EMA), has been proposed by Willoughby & Brickman [52]. Similar to EMA methods that collect real-time data from participants about their experiences, the EMA-style message testing approach developed by Willoughby & Brickman collects data on participants’ perceptions of different candidate health messages using brief surveys sent at multiple times throughout the day over the course of several days [52]. In a feasibility test of this approach with a group of college students assessing sexual health messages, the completion rate for the EMA-style surveys was 91.5%, and self-report and platform metrics suggested that each short survey (consisting of five Likert scale items) took participants less than two minutes to complete (approximately 30 seconds on average) [52]. Additionally, most participants (78%) found the surveys easy to complete, and a majority (64%) said they would prefer completing the mobile-based short surveys over filling out a one-time, longer survey [52]. The authors highlight a few key advantages of this approach, including the fact that the short EMA-style surveys may produce more reliable data (due to reductions in memory errors and fatigue) [52]. Furthermore, EMA-style surveys delivered by mobile phones can increase external validity: message testing is frequently done in a lab setting or using online surveys, but the ubiquity of mobile devices gives researchers the opportunity to access participants and collect data in more naturalistic environments [52]. This method may be especially useful for health communication interventions that will eventually be delivered on mobile devices (e.g., via text): data collected in real time under similar conditions to the eventual intervention may be more reflective of real-world conditions, including changes in mood, competing priorities, distractions, etc. [52]. Although the authors tested this EMA-style approach using mobile devices, it should also be possible to replicate this approach using, for example, social media, if that is where the eventual intervention or campaign will be delivered.

#### Ethical and Practical Considerations

Although these rapid message testing approaches offer a potentially more efficient way to collect data on how the target population perceives candidate messages, there are some notable limitations to these approaches that should be acknowledged. For example, measures of perceived message effectiveness are not perfect proxies for behavior change and may not always predict outcomes in actual effectiveness studies. However, using validated measures of message perceptions can allow inferences to be made about relative effectiveness of candidate messages, enabling campaign developers to quickly and efficiently identify the most promising messages for further testing or deployment [50].

In regard to the EMA-style message testing method specifically, although this approach appears feasible in a young adult population, more work is needed to determine whether other population groups (e.g., older adults) will find this protocol similarly easy to understand and complete. Furthermore, the fact that data are collected in a more “naturalistic” environment may increase external validity of results, but the lack of control over the participant’s context also means that results may be an artifact of irrelevant external variables. Finally, using mobile devices for research studies requires consideration of privacy. For example, Willoughby & Brickman note that if participants had the “preview” function enabled on their phones, the sexual health-related messages being tested could have appeared briefly on their screens (and could have been visible to others nearby) [52].

### 3.3. Rapid Cycle, Iterative Message Testing

A significant barrier to efficient, timely, and agile health communication practice is that it can take many years for communication research to be conducted and results to be disseminated. Many existing message development and testing processes are not rapid enough to be useful to practitioners and are not nimble enough to be responsive to changing conditions [53]. Given the speed at which public discourse changes in the age of 24-hour news cycles and social media, this lack of agility can hamper efforts to effectively integrate evidence into health communication efforts, especially when responding to emergencies or other time-sensitive issues (e.g., changes in public health policies or medical guidelines). Methods that enable researchers to quickly obtain data that can directly inform practice in real time are needed to ensure that health communication efforts, especially those enacted in crisis situations, are evidence-based.

For example, recognizing that policymakers and practitioners needed to quickly identify and deploy effective messages during the COVID-19 pandemic, and that standard message development and testing approaches would not be capable of meeting these needs, Bartels and colleagues drew on agile principles (which focus on incorporating knowledge into practice through rapid creation of usable prototypes based on testing, user feedback, and validation) to develop a 4-step rapid message testing model that would significantly reduce the time needed for evidence-based messages to be put into practice [53]. The steps of the model consist of (1) message creation, (2) survey development, (3) survey administration, and (4) analysis/presentation of results to practitioners (e.g., health department staff), conducted over the course of seven days. Each subsequent cycle of the process is then informed by the previous week’s results [53].

Applying the model to COVID-19 messaging, in Step 1 (days 1 and 2), the team developed messages based on the most recent public health guidance, existing messages, and key theoretical predictors of health behavior change. In Step 2 (days 3 and 4), the message evaluation survey was developed and tested; the survey included the messages developed in Step 1, items to evaluate the messages, and sociodemographic questions. Step 3 (day 5 and 6) involved administering the evaluation survey to approximately 200 members of the target population through MTurk. During Step 4 (days 6 and 7), the survey results were analyzed, interpreted, and presented to key staff at the state department of health. The results included key findings, summary descriptive statistics, message rankings, and differences in message rankings for different subgroups. Insights derived from the surveys could be immediately incorporated by health department staff into public messaging delivered through retail signage and social media. Additionally, after results were presented, health department staff were able to provide feedback and communicate any changes in their needs or priorities, which, along with the results of the prior week’s survey, was used to inform the next iteration of the rapid message testing cycle performed the following week [53].

The model proposed by Bartels et al. has several notable strengths, including its speed (evidence-based recommendations were delivered on a weekly basis) and responsiveness (by working closely with practitioners, researchers were able to ensure that their work would be relevant and useful to the needs of policy makers and public health officials, and the iterative nature of the process enabled the team to make changes as new information emerged and circumstances changed) [53]. The methods used (i.e., MTurk) were also cost-effective and efficient. However, the authors also noted several limitations to their approach, including the fact that the samples used were not representative (the use of consumer panels or crowdsourcing platforms present a tradeoff between generalizability and rapid, convenient data collection) [53]. Nonetheless, additional research testing this model across a wider array of health issues could help advance the field, as this approach presents an innovative model for ensuring that practitioners have timely access to evidence-based messages and that interventions are responsive to a rapidly changing ecosystem.

#### Ethical and Practical Considerations

Relying on digital platforms to conduct rapid data collection may be necessary given the compressed timeline of this approach, but it risks leaving out populations who may be less digitally connected, thereby overlooking their needs and views. Since the digital divide tends to disproportionately affect vulnerable or underserved populations—such as those who are older or have a lower socioeconomic status [54]—this is an important consideration. Researchers can take additional steps to reach those who might not be online, for example by partnering with community-based organizations or recruiting at venues frequented by these groups and integrating the results of those surveys with results from online panels in order to ensure that the voices and needs of vulnerable groups are not overlooked.

Another concern is that this message evaluation protocol is time and resource-intensive, and it may not be feasible to replicate this approach for all health issues a given community is facing, due to limited funding, time, and staffing available to both research teams and health departments. Additionally, the approach focuses on collecting and analyzing quantitative data (e.g., ratings of perceived effectiveness of messages on a Likert scale); adding qualitative data could yield richer and more nuanced results, but qualitative data analysis may be challenging to incorporate into the protocol given the compressed time frame.

### 3.4. Megastudies

Another barrier to the translation of research into practice is that typically, different intervention ideas are tested in different samples using different measures over different time intervals, and the consequent heterogeneity in demographics, treatment and follow-up periods, contexts, and outcomes limits the ability to make comparisons of intervention efficacy and inform decision makers about the best course of action [55]. To address these limitations, Milkman and colleagues have proposed the idea of a megastudy—a massive field experiment in which the effects of many different interventions are tested synchronously in one large population using a common, objectively measured outcome [55], an approach that could have many applications in the field of health communication.

For example, Milkman et al. conducted two megastudies to test the most effective text-based nudges for encouraging flu vaccination in different settings [56,57]. The first study, which was conducted with 689,693 Walmart pharmacy customers, tested 22 different text message reminders based on a variety of different behavioral science principles (e.g., establishing social norms, increasing goal commitment) that varied across several different attributes (e.g., interactivity, length, frequency) [54]. Results indicated that the most effective approaches included telling patients that a flu shot was “waiting” for them and sending multiple reminders [56]. The second study tested 19 different text messages in the patient population of two large health systems (N = 47,306), including messages containing videos, quizzes or jokes [57]. Findings suggested that interventions were most effective when they were framed as reminders to get flu shots that were already reserved for the patient and when they were congruent with the sort of communication patients expected from their healthcare provider (i.e., not surprising, casual, or interactive) [57].

Similar megastudies could be used to test a greater range of communication strategies, in additional settings, across a wider array of topics. For example, megastudies could be used to test different misinformation correction approaches on social media (e.g., humor-based correction, logic-based correction, expert correction, peer correction), assess different formats for patient education materials in safety net clinics (e.g., narratives, animated videos, graphic novels), or compare different approaches to communication training in medical schools (e.g., didactic lectures, role play, case study discussions, individual coaching). Because megastudies decrease the marginal costs of conducting field research for individual research teams and accelerate the pace of scientific discovery [55], they could be an important tool for advancing health communication research, and because megastudies make it easier to compare different interventions that have been tested in real-world settings, they can help facilitate the use of research results in practice.

#### Ethical and Practical Considerations

Despite numerous advantages, the megastudy approach also entails several unique considerations. For example, very large sample sizes are required to test multiple interventions with adequate statistical power [55]. Additionally, the effect sizes of top-performing interventions will typically be overestimated whereas the effect sizes of the worst-performing interventions will typically be underestimated due to noise and mean reversion [55].

It is also important to carefully consider participant inclusion and consent processes with these types of projects. For example, several of the text-message-based megastudies that have been conducted included all eligible individuals who did not opt out of receiving messages or who generally agreed to receive communications from the healthcare facility or pharmacy that served as the setting for the study (e.g., [57]). Even though text messages are generally a low-risk intervention, there could be situations where the messages would be problematic or distressing (e.g., sending weight-loss information to someone with a history of disordered eating). In these cases, it may be helpful to allow participants to opt in to receiving messages related to specific topics prior to launching the study or to opt out of participating in a particular study. Moreover, there can be important differences in public attitudes toward opt-in or opt-out strategies [58]. It may therefore be important to carefully weigh the risks and benefits of these strategies for each study—for example, informed consent processes or opt-in approaches may be more burdensome in these larger studies and may also risk reducing the number of individuals who participate (which can affect statistical power), but they could also help ensure that studies have less potential to adversely impact the welfare of participants.

Finally, although one of the most appealing aspects of megastudies is their potential to inform practice because they are conducted in real-world contexts, these settings can also place limitations on the research design. For example, researchers might be restricted to using the communication tools and resources that are available in the healthcare facility where the study is taking place. Additionally, researchers are limited to the populations that can be reached in these settings, and whether this might introduce bias needs to be carefully considered when results are being interpreted. For example, due to where large chain pharmacies choose to locate for business reasons, might their customers differ from individuals who use small independent pharmacies? These aspects will be important to consider when implementing and interpreting megastudies that leverage real-world settings.

### 3.5. Agent-Based Modeling

Given that communication is a complex, multilevel phenomenon, the application of system science methods (a broad class of analytical approaches that aim to uncover the behavior of complex systems [59]) could help further health communication research and practice. For example, agent-based modeling could advance understanding of mechanisms underlying emergent patterns at the group and population level, offer explanations for empirically observed patterns, and generate predictions for hypothetical or unprecedented scenarios [60]. Simulation models were widely used during the COVID-19 pandemic to forecast the trajectory of infections and assess the impact of specific policies, allowing policymakers and public health officials to make more informed decisions about mitigation measures [60]; creating analogous models to help health communication practitioners develop more effective communication plans could be similarly beneficial.

Agent-based modeling is a method for modeling and simulating complex systems based on theoretical principles that uses data for calibration purposes and conceptual validation [60]. The models consist of a collection of agents (with given attributes) that interact with each other in a given environment and follow a set of rules that encode behavioral mechanisms [60]. During the simulation, key parameters are systematically tuned to determine the extent to which these changes affect the results and overall dynamics of the system [60]. The focus of agent-based models is on the individual micro-processes that generate patterns observed on the macro level; however, despite the fact that many research questions in communication science focus on emergent macro phenomena (e.g., echo chambers and polarization) that are well suited to this type of analysis, agent based modeling is underutilized in communication research [60].

The potential usefulness of agent based modeling for studying complex communication phenomena is demonstrated by the work of Geschke et al. [61], who built a model and analyzed 12 different information filtering scenarios to assess the circumstances under which social media algorithms contribute to echo chambers. Noting that information filtering processes can take place at the individual (cognitive motivational processes), social (network structures), and technological (algorithms/recommender systems) levels, the researchers found that even without any social or technological filters, echo chambers still emerge as a consequence of cognitive mechanisms (e.g., confirmation bias) under conditions of central information propagation; however, the addition of social and technological filtering mechanisms results in even more distinct and less interconnected echo chambers [61]. Interestingly, the model indicated that the recommendation of information that is outside an individual’s “latitude of acceptance” (farther away from their pre-existing attitudes) could delay the creation of echo chambers for a time, suggesting that enabling serendipity in recommender systems could attenuate the emergence of echo chambers (at least to an extent) [61], which points to a possible mitigation measure that could be enacted at the technological level. This illustrates how insights from these types of models can be translated into policy recommendations for social media platforms or other actors in the communication environment [60].

In the context of health communication specifically, Barbrook-Johnson et al. [62] used agent-based modeling to explore how simulated individuals would respond to influenza epidemic messages based on their attitudes, epidemic proximity, and other relevant factors. The authors note that communication is one of the few tools available to public health agencies during an epidemic, but that developing effective communication strategies in this context is made difficult by the unpredictable timing and nature of new epidemics and the lack of rigorous empirical research on the effects of communication efforts, which agent-based models can help address [62]. The goal of the model built for the Tell Me Project was to compare the potential effects of different communication plans on protective personal behavior, and consequently on the spread of flu, during an influenza epidemic. This essentially required the linkage of (1) a behavioral model simulating how individuals respond to communication and make decisions about adopting protective behaviors (e.g., vaccination), and (2) an epidemic model that simulates the spread of influenza [62]. In this model, each message tested is constructed from pre-determined choices regarding timing (e.g., every 10 days), targeting (media channel and population group) and content. Simulated individuals respond to messages based on message content characteristics, which can take the values of: (1) promoting benefits, (2) providing epidemic status information, (3) emphasizing norms, and (4) recommending adoption of behaviors [62]. The messages influence the individual’s decision to either adopt or discontinue protective behavior by changing factors that contribute to that decision (e.g., attitudes, threat perceptions) [62]. Although this model used a simplified conceptualization of communication and was non-predictive, stakeholders felt that it could still be useful as a teaching tool to help illustrate the complexity of communication effects during epidemics, as a strategy for identifying weaknesses in theory, and for informing data collection efforts [62]. Additional empirical data regarding individual behaviors and responses to communications could help improve parameterization (the process of defining parameter values) of agent based models designed to simulate communication processes or forecast the impact of communication efforts [62].

#### Ethical and Practical Considerations

Although agent-based modeling has the potential to generate important insights and inform health communication efforts, there are several notable challenges to applying this method that need to be considered. First, it can be difficult to find a balance between model generalization and specificity: models that are too general may not be of practical use, while models that are too specific to a particular context may not be applicable to other situations [60]. Second, it can be challenging to select the appropriate level of model complexity: a model needs to incorporate sufficient complexity to generate new insights, but excessive complexity can limit meaningful interpretation [63]. Working with stakeholders and partners to identify the essential elements needed for the model to be credible and useful could be one way to balance these competing demands [63].

A third challenge concerns the availability and quality of data for model parameterization. The input data are fundamental to the results produced by the model [64], but empirical data are often lacking, particularly for the aspects that are most unique and important to agent based modeling, such as the interactions between units [63]. The availability of data on communication process and outcomes can also be a challenge for agent-based models focused on health communication specifically. For example, for the Tell Me Project, the research team had sufficient data to model health behaviors, such as hand washing, but lacked needed data on responses to communication [65]. Additionally, the data that are available can introduce error and bias into the model, such as when the data are not representative [64]. Data often come from observational studies conducted in a specific population, which may differ in important ways from the target population being modeled [63]. Ethical issues can arise from simulations of reality that are based on biased data; for example, researchers may inadvertently stereotype agents in their model that represent certain groups of people. Researchers must be mindful of the social implications of their simulations and the possibility that the data underlying their model could be biased, particularly if findings will be used to inform policy decisions [66].

A fourth challenge concerns the significant logistical hurdles to successfully applying this approach in health communication. In order to build these models, health communication researchers will likely require substantial training to develop a deep understanding of the method as well the practical skills needed to program these models [67]. The significant time and computing resources needed to develop, run, and validate these models is also a barrier to their widespread use [63].

Finally, it is important to note that the models resulting from this process are not a faithful reflection of the real world. These simulations can serve as a “proof of concept”, but should not be considered conclusive evidence on their own or a substitute for empirical data [66,67]. However, agent-based modeling is a valuable addition to existing research methods and can help answer unique questions that other methods are less suited to. In fact, these models can be especially powerful when combined with empirical methods. Empirical data can be used to validate a model and ensure that it is aligned with observed phenomena, while the model can suggest explanations for observed effects by simulating the specific dynamics underlying the aggregated behavioral patterns found in the empirical data [60].

## 4. Conclusions

In this paper we highlighted several innovative approaches that can help ensure that health communication research and practice is more timely, responsive, and effective (see Table 1 for a summary of included approaches). In the first section, we discussed methods that can be used to deliver health messages and interventions that are more relevant to the unique characteristics and circumstances of an individual or a group. In the second section, we discussed methods that can make health communication research more responsive to the needs of decision makers and campaign designers, thereby reducing the research-to-practice gap. However, although these approaches appear promising, additional work to refine and advance these methods is needed; for example, the theoretical underpinnings and mechanisms of effect for many of these approaches have not yet been adequately explored and a better understanding of how and why these approaches work could help optimize the use of these methods in future efforts and help ensure that interventions leveraging these approaches are theoretically informed, replicable, and ultimately scalable. There is also a need for additional research to generate a more robust evidence base in support of these methods, which would help encourage their use. Larger trials demonstrating the impact of efforts leveraging these approaches on behaviors and health outcomes are needed, as much of the extant work in some of these areas relies on measures of reach, engagement, or perceptions to assess impact, and in other areas, the evidence base is limited to a small number of studies or relatively modest pilot projects.

Furthermore, while these approaches have significant potential, there are also several important issues that should be considered when applying these methods. For example, digital tools—from social media platforms to mobile devices—offer practitioners new ways to identify and deliver effective health messages to target audiences and offer researchers new ways to rapidly and efficiently collect data. However, the digital divide and differing levels of digital literacy mean that not everyone has equal access to the platforms and technologies that some of these novel intervention and research methods rely on (e.g., smartphones, online crowdsourcing platforms, social media). Therefore, the potential of these novel approaches to negatively impact equity needs to be carefully considered. On the other hand, it should be noted that many of these approaches could be effectively deployed in underserved populations (as demonstrated by Bonnevie et al. [18] as well as Faro et al. [27]), and therefore have the potential to reduce disparities as well. The utility, acceptability, and efficacy of these approaches among different subgroups should be further explored to both identify opportunities for advancing equity and detect the potential of exacerbating disparities.

Another important issue for researchers and practitioners to keep in mind is that the use of some of these technologies and methods may entail the loss of a certain degree of control over the process, or the imposition of certain limitations. For example, efforts that rely on social media platforms must comply with the companies’ policies. As Arigo et al. note, there are many advantages to using commercial platforms, but there are also notable drawbacks including lack of clarity around data access and control, the possibility of unexpected changes to policies or technology features, and the potential for practices that do not conform to ethical standards adhered to by health communication professionals and researchers [32]. Therefore, when using commercially available technologies, researchers need to be very familiar with the tools they are using, stay abreast of changes, and inform participants of any new potential risks they might be exposed to [32].

Practitioners and researchers may also face issues related to transparency and informed consent in using these methods. When campaigns or A/B testing studies are conducted on social media platforms, it may not be feasible to collect informed consent from users, and users may not be aware that they are being targeted based on data the platforms have collected. Even when users are explicitly enrolled in a study or intervention, heightened transparency could be appropriate, given the amount of data that may need to be collected or the complexity of the intervention (e.g., in the case of adaptive interventions or recommender systems). It is important that participants fully understand how data are being collected and used. It is also vital that these data are carefully protected as not to violate participant privacy. To the extent possible, researchers should seek to minimize any potential harms or ethical concerns (e.g., by making processes more transparent and ensuring data are collected in ways that respect privacy).

There are also significant practical concerns related to resource availability and feasibility that must be considered. Some of the methods mentioned in this article can help make health communication research and practice processes more efficient and cost-effective (e.g., social media campaigns are relatively cheap); however, other methods, such as adaptive interventions, agent-based modeling, and megastudies, may be highly complex and potentially resource-intensive. Furthermore, since many of the methods discussed are relatively new and most are reliant on technology, practitioners and researchers may also need to invest in training and/or new tools before they can successfully deploy these approaches in their work. However, given the potential of these methods to advance health communication research and practice, and the clear need for innovations in the field, the required investments may be justified.

## Figures and Tables

**Table 1:** Innovative health communication approaches and selected examples of studies leveraging these methods.

Approach	Description	Selected Examples from the Literature
Digital Segmentation and Microtargeting	Efforts that use digital platforms to granularly segment and target audiences based on factors such as demographics, interests, social network characteristics, location, and online behaviors.	Morrison, L.; Chen, C.; Torres, J.; Wehner, M.; Junn, A.; Linos, E. Facebook advertising for cancer prevention: a pilot study. *The British Journal of Dermatology* **2019**, *181*, 858. de Vere Hunt, I.; Dunn, T.; Mahoney, M.; Chen, M.; Nava, V.; Linos, E. A Social Media-Based Public Health Campaign Encouraging COVID-19 Vaccination Across the United States. *American Journal of Public Health* **2022**, *112*, e1–e4. Graham, J.E.; Moore, J.L.; Bell, R.C.; Miller, T. Digital marketing to promote healthy weight gain among pregnant women in Alberta: an implementation study. *Journal of Medical Internet Research* **2019**, *21*, e11534.
Social Media Influencer Campaigns	Campaigns that utilize influencers (i.e., individuals who have a high number of followers on social media, either in general or among a specific subpopulation) to disseminate health messages.	Bonnevie, E.; Rosenberg, S.D.; Kummeth, C.; Goldbarg, J.; Wartella, E.; Smyser, J. Using social media influencers to increase knowledge and positive attitudes toward the flu vaccine. *PLoS ONE* **2020**, *15*, e0240828. Boon, C.; Golloub, E. How social media influencers helped the NYC public health system raise awareness of COVID-19 testing among historically disadvantaged populations. *Journal of Digital & Social Media Marketing* **2021**, *9*, 198–204. Guo, M.; Ganz, O.; Cruse, B.; Navarro, M.; Wagner, D.; Tate, B.; Delahanty, J.; Benoza, G. Keeping it fresh with hip-hop teens: promising targeting strategies for delivering public health messages to hard-to-reach audiences. *Health Promotion Practice* **2020**, *21*, 61S–71S.
Recommendation Algorithms	Systems that generate personalized suggestions through the use of algorithms that predict a target individual’s reaction to items (e.g., messages) that they have not previously interacted with based on the target individual’s past preferences, the preferences of other similar individuals, or a combination of these.	Kim, H.S.; Yang, S.; Kim, M.; Hemenway, B.; Ungar, L.; Cappella, J.N. An experimental study of recommendation algorithms for tailored health communication. *Computational Communication Research* **2019**, *1*, 103–129. Sadasivam, R.S.; Borglund, E.M.; Adams, R.; Marlin, B.M.; Houston, T.K. Impact of a collective intelligence tailored messaging system on smoking cessation: the Perspect randomized experiment. *Journal of Medical Internet Research* **2016**, *18*, e6465.
Adaptive Interventions	Interventions that allow for personalized tailoring of intervention components to better meet an individual’s needs and circumstances.	Dorsch, M.P.; Cornellier, M.L.; Poggi, A.D.; Bilgen, F.; Chen, P.; Wu, C.; An, L.C.; Hummel, S.L. Effects of a novel contextual just-in-time mobile app intervention (LowSalt4Life) on sodium intake in adults with hypertension: pilot randomized controlled trial. *JMIR mHealth and uHealth* **2020**, *8*, e16696. Conroy, D.E.; Hojjatinia, S.; Lagoa, C.M.; Yang, C.-H.; Lanza, S.T.; Smyth, J.M. Personalized models of physical activity responses to text message micro-interventions: a proof-of-concept application of control systems engineering methods. *Psychology of Sport and Exercise* **2019**, *41*, 172–180.
A/B Testing	A strategy for comparing two (or more) versions of a variable through a controlled experiment to assess which option more effectively achieves a pre-specified outcome.	Ziegenfuss, J.Y.; Renner, J.; Harvey, L.; Katz, A.S.; Mason, K.A.; McCann, P.; Mettner, J.; Nelson, K.D.; Taswell, R.; Wacholz, B.K. Peer Reviewed: Responses to a Social Media Campaign Promoting Safe Fish Consumption Among Women. *Preventing Chronic Disease* **2019**, *16,* E99. Fan, K.L.; Black, C.K.; DeFazio, M.V.; Luvisa, K.; Camden, R.; Song, D.H. Bridging the knowledge gap: an examination of the ideal postoperative autologous breast reconstruction educational material with A/B testing. *Plastic and Reconstructive Surgery* **2020**, *145*, 258–266. Austrian, J.; Mendoza, F.; Szerencsy, A.; Fenelon, L.; Horwitz, L.I.; Jones, S.; Kuznetsova, M.; Mann, D.M. Applying A/B testing to clinical decision support: rapid randomized controlled trials. *Journal of Medical Internet Research* **2021**, *23*, e16651.
Efficient Message Testing Protocols	Methods that enable candidate messages to be evaluated quickly and efficiently.	Kim, M.; Cappella, J.N. An efficient message evaluation protocol: two empirical analyses on positional effects and optimal sample size. *Journal of Health Communication* **2019**, *24*, 761–769. Willoughby, J.F.; Brickman, J. Adding to the message testing tool belt: Assessing the feasibility and acceptability of an EMA-style, mobile approach to pretesting mHealth interventions. *Health Communication* **2021**, *36*, 1260–1267.
Rapid Cycle, Iterative Message Testing	A method for quickly and iteratively developing, testing, and sharing messages with practitioners that reduces the time needed for evidence-based messages to be put into practice.	Bartels, S.M.; Gora Combs, K.; Lazard, A.J.; Shelus, V.; Davis, C.H.; Rothschild, A.; Drewry, M.; Carpenter, K.; Newman, E.; Goldblatt, A. Development and application of an interdisciplinary rapid message testing model for COVID-19 in North Carolina. *Public Health Reports* **2021**, *136*, 413–420.
Megastudies	Large field experiments in which the effects of many different interventions are tested synchronously in one population using common, objectively measured outcomes.	Milkman, K.L.; Gandhi, L.; Patel, M.S.; Graci, H.N.; Gromet, D.M.; Ho, H.; Kay, J.S.; Lee, T.W.; Rothschild, J.; Bogard, J.E. A 680,000-person megastudy of nudges to encourage vaccination in pharmacies. *Proceedings of the National Academy of Sciences* **2022**, *119*, e2115126119. Milkman, K.L.; Patel, M.S.; Gandhi, L.; Graci, H.N.; Gromet, D.M.; Ho, H.; Kay, J.S.; Lee, T.W.; Akinola, M.; Beshears, J. A megastudy of text-based nudges encouraging patients to get vaccinated at an upcoming doctor’s appointment. *Proceedings of the National Academy of Sciences* **2021**, *118*, e2101165118.
Agent-Based Modeling	A method for simulating complex systems, where autonomous agents (whose actions are governed by a set of rules that encode behavioral mechanisms) interact with each other in a given environment.	Barbrook-Johnson, P.; Badham, J.; Gilbert, N. Uses of agent-based modeling for health communication: The TELL ME case study. *Health Communication* **2017**, *32*, 939–944.

## Data Availability

Not applicable.
